# Altered EEG microstate associated with anxiety and somatization symptoms in major depressive disorder

**DOI:** 10.3389/fpsyt.2026.1772171

**Published:** 2026-03-06

**Authors:** Qinfan Shan, Guisen Wu, Yuxuan Xiong, Qian Guo, Hao Hu, Fuxu Zhang, Zhenying Qian, Tianhong Zhang, Xiaohua Liu

**Affiliations:** 1 Department of Psychiatry, Shanghai Mental Health Center, Shanghai Jiao Tong University School of Medicine, Shanghai, China; 2 Shanghai Key Laboratory of Psychotic Disorders, Shanghai Mental Health Center, Shanghai Jiao Tong University School of Medicine, Shanghai, China

**Keywords:** anxiety, EEG microstates, major depressive disorder, resting-state EEG, somatization

## Abstract

**Objective:**

To examine abnormalities in EEG microstate dynamics in patients with major depressive disorder (MDD) and to explore their associations with anxiety and somatization symptoms.

**Methods:**

We enrolled 30 patients with MDD and 40 healthy controls. Resting-state EEG was recorded and analyzed using microstate segmentation (classes A–D). Temporal parameters (mean duration, occurrence, time coverage, and transition probabilities) were compared between groups, and correlations with clinical symptoms (HAMD, HAMA, MADRS) were examined.

**Results:**

Compared with controls, patients with MDD exhibited a significantly longer duration, higher occurrence, and greater time coverage of microstate C, while microstate B showed reduced occurrence and coverage. Transition probability analyses revealed fewer transitions from A to B, A to D, B to A, B to D, and D to A, and more transitions between C and D. Symptom correlations indicated that microstate B occurrence was positively associated with HAMD anxiety/somatization scores, while transitions from C to D and from D to C were negatively correlated with anxiety/somatization scores.

**Conclusions:**

MDD is characterized by alterations in microstate B and C dynamics and disrupted transitions between C and D, some of which relate to symptom dimensions, suggesting that EEG microstate features may serve as potential neurophysiological markers in major depressive disorder.

## 1 Introduction

Major depressive disorder (MDD) is a common and serious mental disorder characterized by persistent depressed mood, loss of interest, and a range of cognitive and somatic symptoms, which significantly impair the quality of life and social functioning (1). Epidemiological studies indicated that its prevalence and disease burden have continued to rise over recent decades (2). According to the latest Global Burden of Disease Study (GBD 2021), depression has become one of the leading causes of disability, affecting more than 300 million people worldwide (3). MDD is recognized as a heterogeneous disorder involving genetic vulnerability, neurochemical dysregulation, and environmental stressors (4). Previous neuroimaging and neurobiological studies have provided important perspectives on the structural and functional abnormalities associated with MDD (5). However, the temporal dynamics of large-scale brain activity remain unclear.

Electroencephalography (EEG) is a noninvasive technique with high temporal resolution that allows the assessment of neural dynamics on a millisecond timescale (6). EEG microstates were first introduced by Lehmann (7), who described them as short periods of quasi-stable topographies, typically lasting between 60 and 120 ms, and considered them to be the basic building blocks of human mentation. Unlike conventional spectral or event-related potential analyses that focus on single-channel or frequency-specific activity, microstate analysis simultaneously considers the signal from all electrodes to generate a global representation of brain activity. This approach captures the rapid sequence of scalp topographies that occur over milliseconds, providing a unique window into spontaneous cognitive processing. The brief, quasi-stable patterns are believed to reflect coordinated neural activity involving large-scale, spatially distributed brain networks.

Subsequent studies confirmed that four canonical microstate classes can be reliably identified, explaining the majority of variance in resting-state EEG recordings, which are conventionally labeled as microstates A, B, C, and D (8). These canonical microstates exhibit distinct and reproducible scalp topographies and have been linked to different large-scale functional systems based on EEG–fMRI and source localization evidence (9, 10). In general, microstates A and B are associated with sensory processing networks, such as auditory and visual systems (11), whereas microstate C has been linked to the salience network and self-referential processing, and microstate D is generally related to attentional control and executive networks (12).

Temporal parameters of microstates, such as mean duration, time coverage, occurrence, and transition probabilities (13), provide a framework for quantifying the dynamic coordination of large-scale brain activity.

Previous studies have applied EEG microstate analysis to a range of psychiatric disorders, such as schizophrenia (14), bipolar disorder (15), and obsessive compulsive disorder (16), revealing that alterations in microstate dynamics may serve as potential neurophysiological markers. In major depressive disorder, emerging evidence also suggests abnormalities in microstate parameters (17), but the reported findings have been heterogeneous. This heterogeneity may partly reflect the intrinsic clinical complexity of MDD and differences in sample characteristics across studies. Importantly, the relationships between microstate alterations and clinical symptoms remain unclear. To address this gap, this study aimed to explore EEG microstate abnormalities in patients with MDD and examine their associations with clinical symptoms. We hypothesized that alterations involving microstate C might be associated with clinical symptom dimensions in patients with MDD.

## 2 Methods

### 2.1 Participants

We enrolled thirty patients diagnosed with major depressive disorder (MDD) from the outpatient clinic of Shanghai Mental Health Center. The diagnosis of MDD was made by qualified psychiatrists according to the Diagnostic and Statistical Manual of Mental Disorders, Fifth Edition (DSM-5). All patients were between 16 and 60 years of age. Individuals were not included if they presented with intellectual disability or other severe psychiatric disorders, including schizophrenia, bipolar disorder, or other psychotic disorders. Participants with a history of suicide attempts or current suicidal risk were also excluded. Additional exclusion criteria were organic neurological disease, serious systemic or metabolic illness, and substance dependence. Individuals with sensory impairments that could interfere with EEG recording, such as severe myopia, hearing loss, or color vision deficiency, were excluded.

The control group comprised forty healthy volunteers recruited through community advertising. They were required to be between 16 and 60 years old and to have completed at least elementary education. All healthy controls were screened by trained psychiatrists using the Mini-International Neuropsychiatric Interview (MINI), version 7.0 (18). Volunteers were excluded if they exhibited current psychological symptoms such as mild depression or anxiety, reported a personal or family history of psychiatric illness, or had sensory impairments similar to those specified for the patient group.

All participants gave written informed consent prior to participation, and consent for those under 18 years was additionally obtained from parents or legal guardians. The study protocol was reviewed and approved by the Ethics Committee of Shanghai Mental Health Center.

### 2.2 Clinical assessment

Clinical symptoms were assessed using the 24-item Hamilton Depression Rating Scale (HAMD-24) (19), the Hamilton Anxiety Rating Scale (HAMA) (20), and the Montgomery–Åsberg Depression Rating Scale (MADRS) (21). Clinical assessments were completed after the EEG recording. For further analysis, the HAMD-24 was divided into seven factors: anxiety/somatization, weight, cognitive impairment, diurnal variation, retardation, sleep disorder, and sense of despair (22).

### 2.3 EEG data acquisition and preprocessing

Resting-state EEG was collected in a quiet, electrically shielded room while participants sat comfortably with eyes closed for 3 minutes. Signals were obtained using a 64-channel cap arranged according to the international 10–20 system (Brain Products GmbH, Gilching, Germany). AFz served as the ground electrode and the nose tip as the recording reference. Data were digitized at 1000 Hz with an online band-pass setting of 0.016–200 Hz, and electrode impedances were kept below 5 kΩ during acquisition. EEG preprocessing was performed in MATLAB 2023b using the EEGLAB toolbox (23). Two electrooculogram channels (IOLeft and IORight) were excluded prior to analysis. Continuous EEG data were band-pass filtered between 1 and 40 Hz. Independent component analysis (ICA) was performed (24), and artifactual components such as ocular and muscle activity were identified using the ICLabel plugin with a 90% classification probability threshold and removed after manual confirmation (25). The data were segmented into 2-s epochs, and epochs containing residual artifacts were discarded after visual inspection. Epochs with voltage amplitudes exceeding ±100 μV were excluded. The remaining cleaned EEG data were then re-referenced to the common average reference and used for microstate analysis.

### 2.4 Microstate analysis

EEG microstate analysis was performed using Cartool software (26). Preprocessed EEG data were and band-pass filtered between 2 and 20 Hz prior to microstate segmentation.

Global field power (GFP) was defined as the standard deviation of the potentials across all electrodes of an average-reference map (27). EEG scalp maps at GFP peaks were extracted for microstate analysis, as these time points are characterized by a higher signal-to-noise ratio and reflect more stable scalp topographies. At the individual level, these maps were clustered using topographic atomize and agglomerate hierarchical clustering (T-AAHC) (28). At the group level, individual microstate maps were combined to derive group-level templates. The templates were categorized into four canonical microstate classes (A–D) with reference to prior studies (8). The group-level microstate templates were backfitted to the continuous EEG data of each participant. Temporal post-processing was applied. Microstate segments shorter than or equal to 20 time frames (TFs) were rejected. Mean duration, occurrence, time coverage, and transition probabilities were extracted for each microstate class.

Based on the resulting microstate sequence, temporal parameters were calculated for each microstate class. Mean duration was calculated as the average duration of continuous time periods labeled as the same microstate (29). Time coverage was calculated as the proportion of the total recording time dominated by a given microstate (30). Occurrence was calculated as the number of appearances of a given microstate per second of the recording (17). Transition probability was calculated as the probability of transitioning from one microstate class to another across all observed microstate transitions (7).

### 2.5 Statistical analysis

Group differences in demographic and clinical variables were assessed using independent-sample t tests for continuous variables and chi-square tests for categorical variables. For microstate parameters (mean duration, occurrence, time coverage, and transition probabilities), group comparisons between patients with MDD and healthy controls were conducted using analysis of covariance (ANCOVA), with years of education included as a covariate. P-values were corrected for multiple comparisons using the false discovery rate (FDR) method.

Within the MDD group, Pearson correlation analyses were performed to examine associations between microstate parameters and clinical symptom scores (HAMD-24 total and factor scores, HAMA, and MADRS).All statistical analyses were conducted in R (version 4.4.3), and the significance threshold was set at p < 0.05 (two-tailed).

## 3 Results

### 3.1 Demographic and clinical characteristics

Demographic variables, including age, sex, and years of education, were recorded for all participants, and clinical measures (HAMD, HAMA, and MADRS) were obtained for the patient group. The demographic and clinical data are summarized in **Table 1**. There were no significant differences between the groups in age or sex distribution (both p > 0.05). Only years of education differed significantly between groups, being lower in the MDD group than in controls.

**Table 1:** Demographic characteristics of MDD and HC group. .

Variables	MDD (n = 30)	HC (n = 40)	t/χ² (p value)	Cohen’s d
Age (years)	26.40 ± 7.36	24.10 ± 5.99	1.442 (p = 0.154)	0.35
Sex (male/female)	8/22	16/24	0.826 (p = 0.364)	—
Education (years)	14.30 ± 3.53	15.85 ± 2.55	-2.133 (p = 0.037)	0.52
HAMD-24 total score	23.07 ± 8.29	—	—	—
Anxiety/somatization	5.50 ± 2.61	—	—	—
Weight	0.63 ± 0.85	—	—	—
Cognitive impairment	5.00 ± 2.64	—	—	—
Diurnal variation	0.73 ± 0.64	—	—	—
Retardation	2.37 ± 1.71	—	—	—
Sleep disorder	2.93 ± 1.62	—	—	—
Sense of despair	5.90 ± 2.28	—	—	—
HAMA score	18.47 ± 7.67	—	—	—
MADRS score (n = 29)	22.10 ± 8.67	—	—	—

Values are presented as mean ± SD. MADRS scores were obtained for 29 of the 30 MDD patients. “—” indicates not applicable for the healthy control group.

### 3.2 EEG microstate parameters: mean duration, occurrence, and time coverage

**Figure 1:**
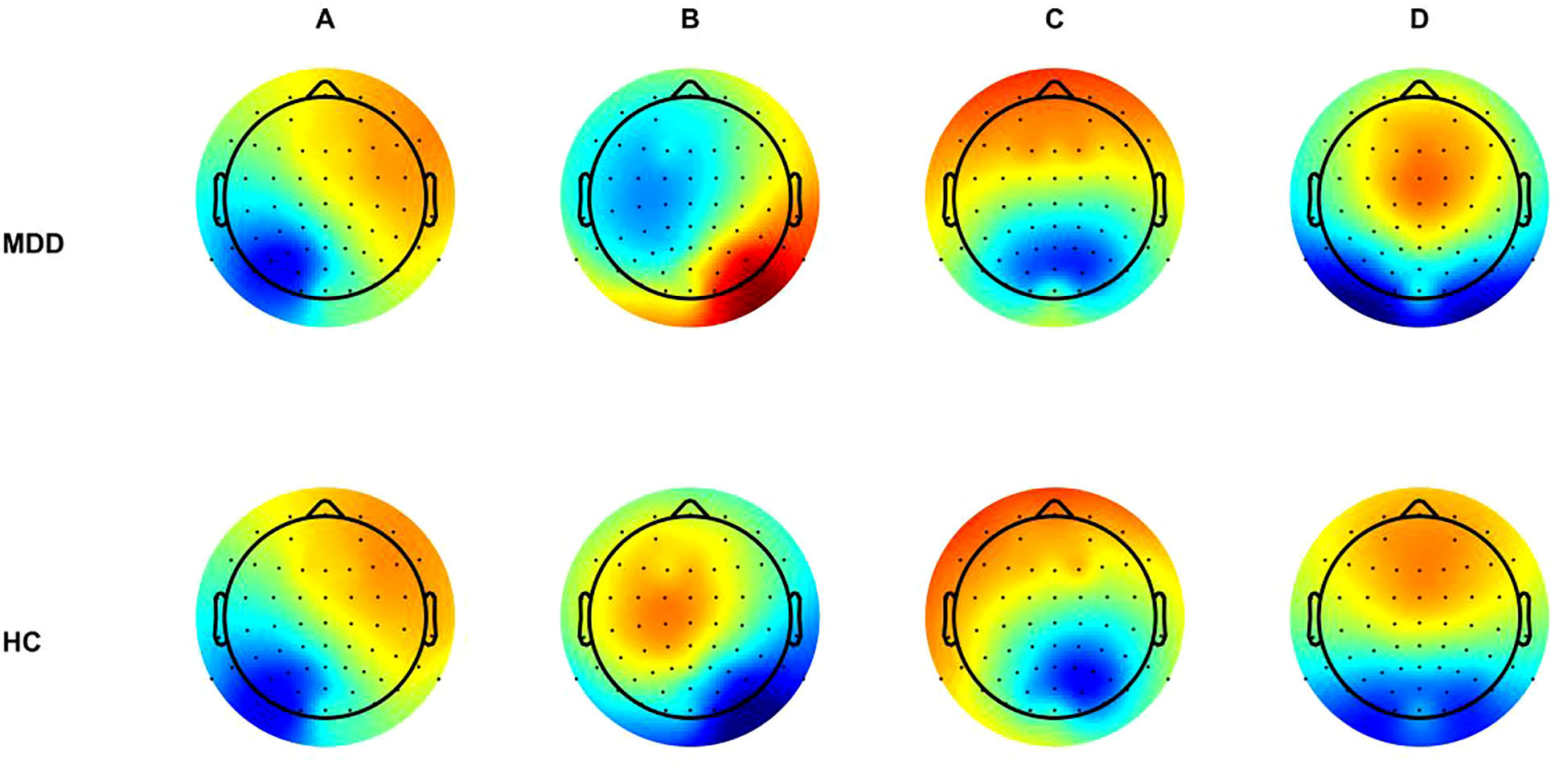
Topographic features of microstates between MDD and HC groups. MDD, major depressive disorder; HC, healthy control; **(A)** microstate A; **(B)** microstate B; **(C)** microstate C; **(D)** microstate D.

**Figure 2:**
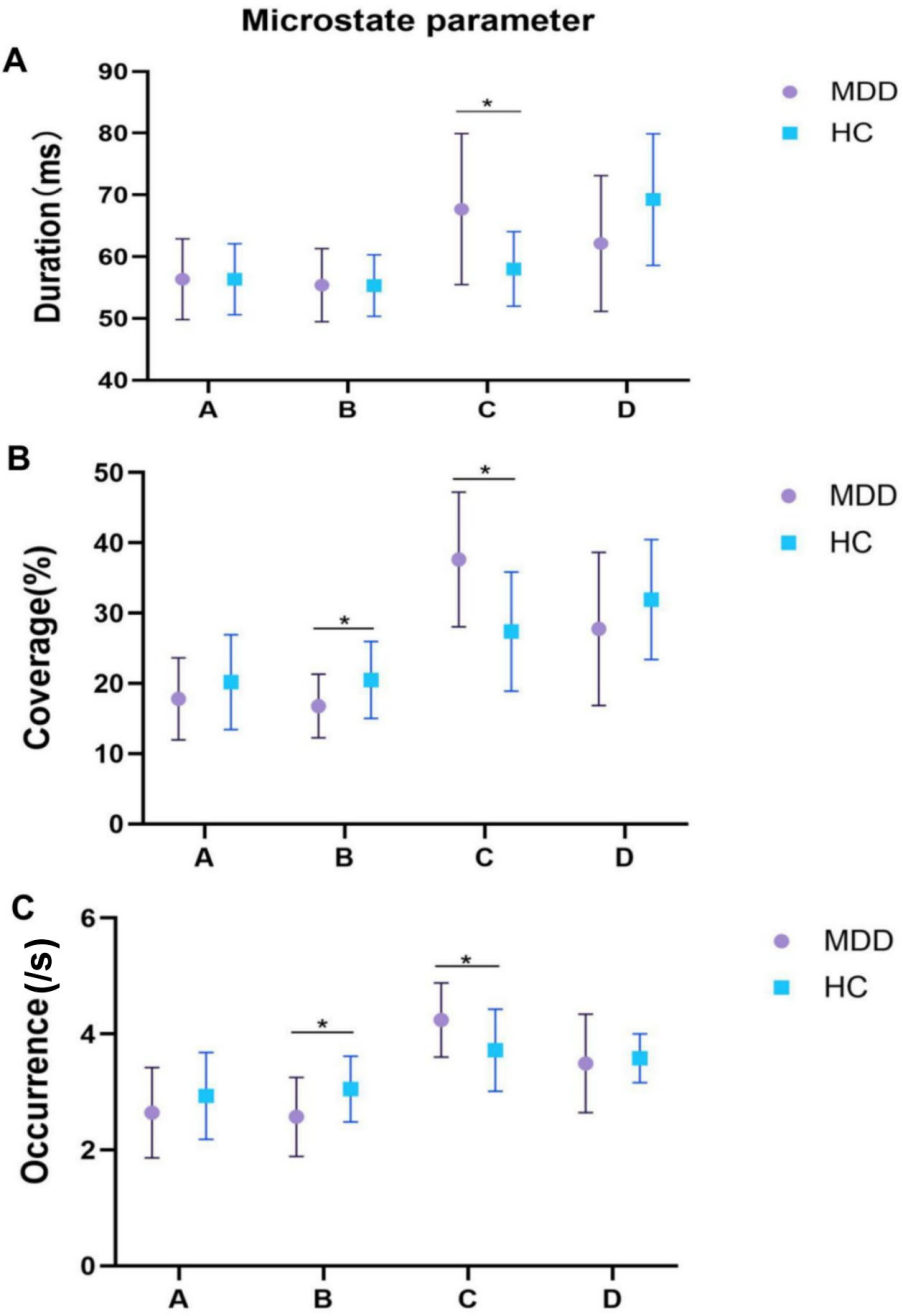
Temporal parameters of microstates (A–D) in MDD and HC. **(A)** Mean duration, **(B)** time coverage, and **(C)** occurrence rate of microstates (A–D) in MDD and HC. * indicates p < 0.05.

Consistent with previous studies, four canonical microstate classes (A–D) were identified in both the MDD and HC groups. Their scalp topographies showed the typical spatial configurations reported in the literature: a right frontal-to-left posterior orientation for microstate A, a left frontal-to-right posterior orientation for microstate B, an anterior–posterior pattern for microstate C, and a fronto-central maximum for microstate D (31) (**Figure 1**). Significant differences in the temporal characteristics of EEG microstates were observed between patients with MDD and healthy controls, as illustrated in **Figure 2**. The MDD group showed a significantly longer mean duration of microstate C compared with healthy controls (MDD: 67.70 ± 12.24 ms; HC: 58.03 ± 6.05 ms; F(1, 67) = 17.46, p = 0.001, η²p = 0.207). No significant differences were observed for microstates A, B, or D in mean duration. Regarding occurrence and time coverage, significant group differences were found for microstates B and C. For microstate B, patients with MDD showed a lower occurrence than healthy controls (MDD 2.57 ± 0.68/s; HC 3.05 ± 0.57/s; F(1, 67) = 10.72, p = 0.007, η²p = 0.138), as well as reduced time coverage (MDD 16.79 ± 4.53%; HC 20.49 ± 5.49%; F(1, 67) = 9.15, p = 0.011, η²p = 0.120). In contrast, microstate C occurred more frequently and covered a larger proportion of time in patients with MDD than in healthy controls (occurrence: MDD 4.24 ± 0.64/s; HC 3.72 ± 0.71/s; F(1, 67) = 7.35, p = 0.020, η²p = 0.099; time coverage: MDD 37.65 ± 9.58%; HC 27.38 ± 8.47%; F(1, 67) = 18.69, p = 0.001, η²p = 0.218). No significant group differences were detected for microstates A or D in either occurrence or coverage.

### 3.3 EEG microstate parameters: transition probabilities

**Figure 3:**
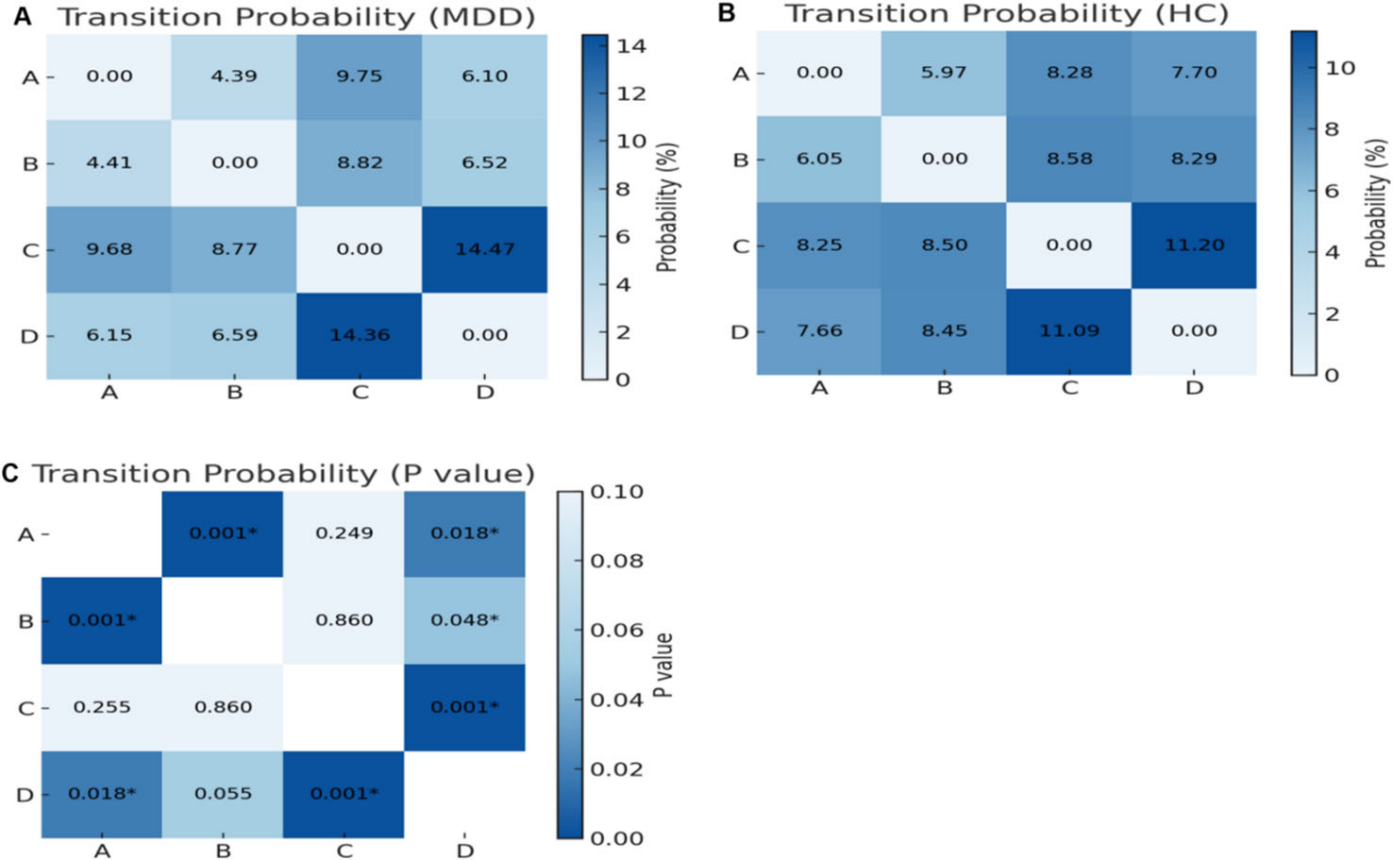
Group differences in transition probabilities between microstates A–D in MDD and HC. **(A)** MDD group, **(B)** HC group, and **(C)** between-group p values. Each cell shows the transition probability from the microstate in the row to the microstate in the column.*p < 0.05.

Group differences in transition probabilities are presented in **Figure 3**. The MDD group had significantly lower transition probabilities from A to B (F(1, 67) = 18.29, p < 0.001, η²p = 0.214), A to D (F(1, 67) = 7.60, p = 0.018, η²p = 0.102), B to A (F(1, 67) = 20.55, p < 0.001, η²p = 0.235), B to D (F(1, 67) = 5.04, p = 0.048, η²p = 0.070), and D to A (F(1, 67) = 7.19, p = 0.018, η²p = 0.097). Higher transition probabilities were observed from C to D (F(1, 67) = 16.97, p < 0.001, η²p = 0.202) and from D to C (F(1, 67) = 15.37, p < 0.001, η²p = 0.187).No other transitions showed significant between-group differences.

### 3.4 Correlations between clinical characteristics and EEG parameters

**Figure 4:**
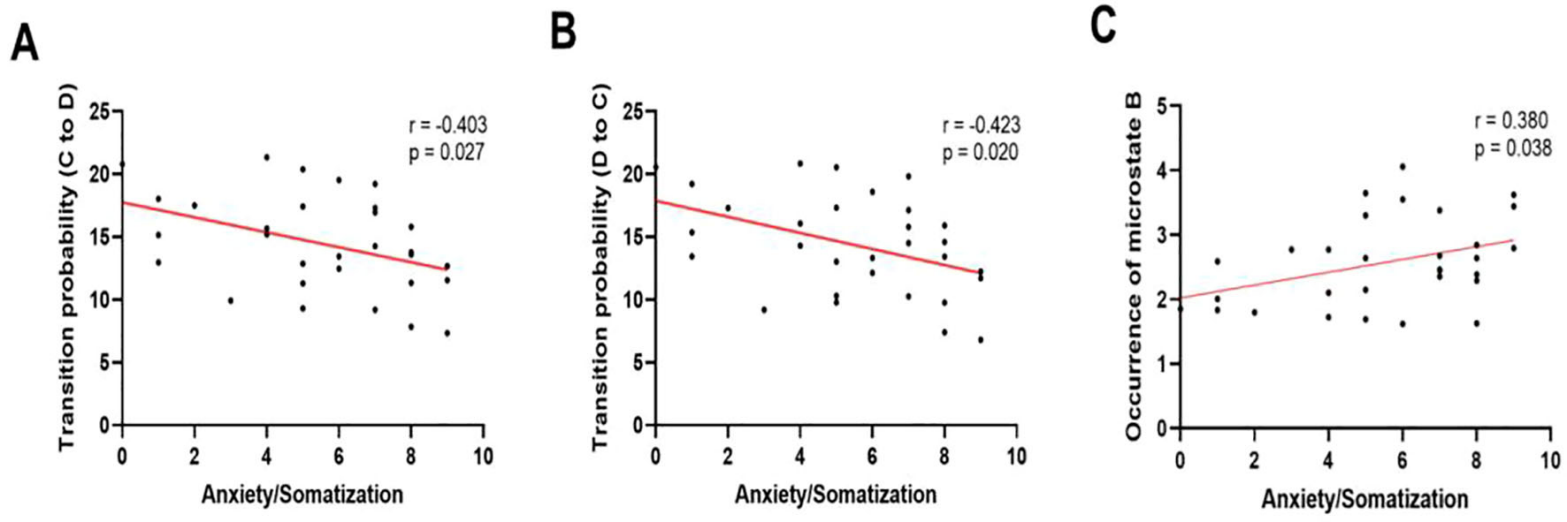
Correlations between anxiety/somatization symptoms and EEG microstate parameters in patients with MDD. **(A)** Correlation between the transition probability from microstate C to D and the HAMD-24 anxiety/somatization factor (r = −0.403, 95% CI = −0.666–−0.050, p = 0.027). **(B)** Correlation between the transition probability from microstate D to C and the HAMD-24 anxiety/somatization factor (r = −0.423, 95% CI = −0.680–−0.074, p = 0.020). **(C)** Correlation between the occurrence of microstate B and the HAMD-24 anxiety/somatization factor (r = 0.380, 95% CI = 0.023–0.651, p = 0.038).

**Figure 4** presents the correlations between microstate transition probabilities and clinical symptom factors in the MDD group. Among the parameters that showed significant group differences, the probability of transition from D to C was negatively correlated with the HAMD-24 anxiety/somatization factor (r = −0.423, p = 0.020). The probability of transition from C to D was also negatively correlated with the HAMD-24 anxiety/somatization factor (r = −0.403, p = 0.027). In addition, the occurrence of microstate B was positively correlated with the HAMD-24 anxiety/somatization factor(r = 0.380, p = 0.038).

## 4 Discussion

In this study, we found that patients with major depressive disorder exhibited longer duration, more frequent occurrence, and increased time coverage of microstate C. Microstate B was reduced in both occurrence and coverage. The analysis of transition probabilities showed more transitions between microstates C and D. Some of these alterations were correlated with clinical symptom severity.

Microstate C represented one of the most prominent alterations observed in the present study. Several previous studies in depression have reported similar trends (32, 33). Microstate C has been functionally linked to the Salience Network (SN), particularly involving the right anterior insula and the posterior part of the anterior cingulate cortex (ACC) (10), and has also been suggested to overlap with the Default Mode Network (DMN). The hyperactivity of microstate C likely reflects an over-engagement with self-referential processes, which are considered neurobiological hallmarks of depressive rumination (34). Conversely, we observed decreased occurrence and time coverage of microstate B in patients with MDD. Given that microstate B has been commonly linked to visual processing, this finding may reflect a withdrawal from external sensory processing. Such a pattern is consistent with reduced environmental engagement in depression.

Interestingly, within the MDD group, microstate B occurrence positively correlated with anxiety/somatization scores. While MDD overall showed lower occurrence and time coverage of microstate B, higher anxiety/somatization levels may necessitate a relative increase in visual-related network activity, potentially demonstrating a state of attentional bias toward perceived threats in individuals with elevated anxiety/somatization symptoms (35). This interpretation receives partial support from a recent meta-analysis of EEG microstates in mood and anxiety disorders, which reported that microstate B occurrence tends to be elevated in patients with comorbid anxiety conditions (36).

A pivotal finding of the present study is that patients with MDD exhibited significantly higher transition probabilities between microstates C and D than healthy controls. Within the MDD group, these transition probabilities were negatively correlated with anxiety and somatization severity. One possible explanation is that the increased transitions between microstates C and D reflect a compensatory mechanism. Microstate D is associated with a fronto-parietal attention network involved in control and reorientation of attention (10, 12). The elevated shifts between microstates C and D may indicate unstable brain dynamics, characterized by more frequent reallocation of cognitive resources between self-referential processes and the external environment in patients with MDD. Meanwhile, previous studies have reported reduced global efficiency in MDD (37). In this context, the increased transitions between microstates C and D may be related to the need to maintain cognitive and emotional functioning. Importantly, longitudinal evidence further supports the above perspective. A recent study showed that transition probabilities between microstates C and D were enhanced at baseline in patients with MDD and decreased following 8 weeks of agomelatine treatment (38). Within the MDD group, this dynamic pattern is not uniformly expressed across different clinical phenotypes. Specifically, in patients with more severe anxiety and somatization symptoms, regulatory dynamics may become constrained, possibly because increased compensatory demands exceed the capacity for flexible large-scale regulation, resulting in reduced flexibility of transitions between microstates C and D.

To sum up, the present study demonstrates that patients with major depressive disorder exhibit distinct alterations in EEG microstate dynamics. Altered transitions between microstates C and D and microstate B occurrence were associated with anxiety and somatization symptom dimensions in MDD. At the same time, it should be noted that EEG microstate analysis may be influenced by analytical approaches and clinical sample characteristics, such as age range and the presence of suicidal ideation (39, 40). Future studies with larger and more diverse samples are warranted to further clarify the neural mechanisms underlying major depressive disorder.

## 5 Limitations

The sample size of this study was relatively small. The cross-sectional design limits the ability to draw causal inferences between EEG microstate alterations and clinical symptoms. Further research with larger samples and longitudinal designs is needed to confirm and extend these findings, which may help improve our understanding of the neural mechanisms underlying MDD.

## 6 Conclusion

This study demonstrated that patients with major depressive disorder exhibit distinct alterations in EEG microstate dynamics, particularly involving microstates B and C. These alterations included increased temporal parameters of microstate C and decreased occurrence and time coverage of microstate B, alongside disrupted transition patterns between microstates. Some of these changes were associated with symptom severity, suggesting that EEG microstate features may serve as potential neurophysiological markers of depression. These findings contribute to a growing body of evidence supporting the utility of EEG microstate analysis in understanding the large-scale brain network dysfunctions underlying depressive disorders.

## Data Availability

The raw data supporting the conclusions of this article will be made available by the authors, without undue reservation.
